# Gut Microbiota Modulation and Anti-Obesity Potential of Epigallocatechin-3-Gallate-Quercetin-Rutin Against High-Fat Diet-Induced Obesity in Rats

**DOI:** 10.3390/life15081331

**Published:** 2025-08-21

**Authors:** Yu-Jou Chien, Ching-Chang Cho, Yu-Ting Hung, Li-You Chen, Yue-Ching Wong, Shiuan-Chih Chen, Chin-Lin Hsu

**Affiliations:** 1 Department of Nutrition, Chung Shan Medical University, Taichung 40201, Taiwan; aa910149@gmail.com (Y.-J.C.); ccjwo21@gmail.com (C.-C.C.); tvickytw7@gmail.com (Y.-T.H.); wyc@csmu.edu.tw (Y.-C.W.); 2 Department of Anatomy, School of Medicine, College of Medicine, Chung Shan Medical University, Taichung 40201, Taiwan; peiyu@csmu.edu.tw; 3 Institute of Medicine and School of Medicine, Chung Shan Medical University, Taichung 40201, Taiwan; sccy399@gmail.com; 4 Department of Family and Community Medicine, Chung Shan Medical University Hospital, Taichung 40201, Taiwan; 5 Department of Nutrition, Chung Shan Medical University Hospital, Taichung 40201, Taiwan

**Keywords:** high-fat diet, anti-obesity, epigallocatechin-3-gallate, quercetin, rutin, gut microbiota

## Abstract

Polyphenols have been widely recognized for their potential anti-obesity effects. This study aimed to evaluate the impact of a polyphenol compound-epigallocatechin-3-gallate, quercetin, and rutin (EQR) on obesity-related parameters and gut microbiota composition. After four weeks of high-fat diet (HFD) induction, the obese Wistar male rats received EQR treatment for an additional four weeks. EQR supplementation significantly reduced body weight gain, feed efficiency, adipose tissue accumulation, and liver lipid content in obese rats. Additionally, it enhanced fecal short-chain fatty acid (SCFA) levels and modulated gut microbiota composition. Specifically, EQR treatment significantly induced *Fusobacteria*, *Fusobacteriaceae*, *Christensenellaceae*, *Christensenellaceae R-7 group*, *Lachnoclostridium*, *Enterorhabdus*, and *Parvibacter* levels and reduced *Deferribacteres* and *Mucispirillum* levels. Gene expression analysis in liver, white adipose tissue (WAT), and brown adipose tissue (BAT) revealed that EQR upregulated the expression of liver *PPAR-α*, WAT *SIRT-1*, and BAT *PGC-1α*, while downregulating liver *PPAR-γ*, liver *FATP-1*, and WAT *FAS*, indicating its role in promoting fatty acid oxidation and thermogenesis, as well as suppressing lipid synthesis and transport. In conclusion, EQR demonstrated significant anti-obesity effects by modulating gut microbiota and lipid metabolism, suggesting its potential as a functional ingredient for obesity management.

## 1. Introduction

Obesity is a chronic disease intricately linked to metabolic syndrome, which predisposes individuals to type 2 diabetes mellitus, cardiovascular disorders, gallbladder dysfunction, cancer, and other comorbidities [1]. The current obesity rate in Europe and the United States exceeds 40% [2]. Prolonged obesity has been shown to reduce quality of life and shorten life expectancy [3]. While pharmacological treatments are available, they often come with adverse side effects. Recent studies indicate that natural compounds hold potential for delivering health benefits and alleviating obesity-related symptoms with minimal side effects. This positions them as a promising focus for ongoing research [4].

Polyphenols, which are abundant in fruits and vegetables, are pivotal components of plant-based diets. Prominent examples include the Mediterranean and Okinawa diets, both of which have been related to a descended risk of metabolic diseases [5,6]. Among these compounds, epigallocatechin-3-gallate (EGCG), a flavonoid derived from green tea that exhibited extreme antioxidant properties, has been shown to promote energy expenditure and decrease body fat accumulation [7]. Similarly, quercetin and rutin, flavonoids commonly present in vegetables, fruits, and seeds, have demonstrated anti-obesity properties. Previous reports indicated that 0.2% quercetin or 10% tartary buckwheat (containing 0.27% rutin and 0.01% quercetin) for 12 weeks exhibited declined body weight, adipose tissue weight, and inflammatory markers in HFD-fed rats [8].

Moreover, polyphenols function as potent antioxidants, thereby providing protective effects on the intestinal tract. Due to their high concentration in the intestinal lumen, estimated to be 100 to 1000 times greater than in the bloodstream, they are well positioned to alter the composition of the intestinal microbiome. This modulation can promote the generation of beneficial metabolites, including bile acids, SCFAs, and trimethylamine N-oxide (TMAO). These metabolites are key regulators of metabolic health [9].

The gut microbiota is a diverse and dynamic community of microbes inhabiting the gastrointestinal tract of humans and animals, which plays a fundamental role in metabolic homeostasis by regulating body fat storage, appetite control, and cytokine production, thereby influencing the development of metabolic diseases [1,10]. An elevated abundance of *Firmicutes* was observed in overweight and obese individuals, which enhance carbohydrate metabolism and increase energy absorption, whereas *Bacteroidetes*, associated with normal body weight, are significantly reduced [11]. These findings suggest a strong connection between gut microbiota composition and obesity. This underscores the potential of microbiota-targeted strategies in obesity prevention and treatment.

Our previous research demonstrated that rutin and quercetin significantly inhibited glycerol-3-phosphate dehydrogenase (GPDH) levels and intracellular triglyceride (TG) contents in 3T3-L1 adipocytes [12]. However, their potential synergistic effects when used in combination have not been thoroughly investigated. To enhance their biological efficacy, we formulated a compound—epigallocatechin-3-gallate, quercetin, and rutin (EQR). The anti-obesity effects of EQR were evaluated using obesity-related markers, gut microbiota composition, and the underlying mechanisms in obese HFD-fed rats.

## 2. Materials and Methods

### 2.1. Sample Composition

The EQR samples are incorporated into a dough composed of the following ingredients: EGCG extracted from American camellia, quercetin derived from onion extract, rutin concentrate powder, flour, and water. In every 103 mg of the EQR supplement, the pure contents of EGCG, quercetin, and rutin were 25 mg, 25 mg, and 1.06 mg, respectively.

### 2.2. Animal Experimental Protocol

The experimental design was based on the studies of Ting et al. [13]. A total of 50 male six-week-old Wistar rats, weighing between 176–200 g, were procured from BioLASCO (Yilan, Taiwan). The entire study received ethical approval from the Animal Care Committee of Chung Shan Medical University (IACUC Approval No: 2026, Approval Date: 25 December 2017). Rats were housed in individual stainless-steel cages under controlled environmental conditions: 50–60% relative humidity, 23 ± 1 °C, while a half-day light/dark cycle. Following a one-week acclimation, the rats were randomly allocated into either a normal diet (ND) group or a high-fat diet (HFD) group using systematic randomization. Our core strategy for reducing confounding factors is to use stratified randomization based on initial body weight to ensure that experimental animals are fairly distributed among the various groups. The dietary formulations aligned with Liu et al. [14], where the ND was a standard AIN-93M diet, and the HFD was modified to increase fat-derived calories from 9.35% to 54.05%, providing energy densities of 3.85 kcal/g and 5.05 kcal/g, respectively. Food and water were provided without restriction. Once the weight difference between the ND and HFD groups exceeded 10%, the HFD group was further divided into four subgroups, receiving either EQR control (0 mg/kg) or low (103 mg/kg), medium (206 mg/kg), or high (514 mg/kg) doses via oral gavage once daily. EQR was administered via gavage to ensure accurate dosing, with procedures conducted gently to reduce stress, though we recognize the potential ethical implications and will consider dietary mixing in future studies. Each group consisted of 10 rats, and the treatment lasted for four weeks. The day before the experiment concluded, the rats fasted for 12 h before being anesthetized using carbon dioxide. Blood samples were collected and analyzed using an automated biochemistry and immunoassay analyzer (C501, Roche, Basel, Switzerland). Organs and tissues were promptly weighed and preserved at −80 °C for subsequent analytical procedures. Mesenteric adipose tissue was carefully cut away from the mesentery along the outer edge of the intestines. Retroperitoneal adipose tissue was obtained by separating the skin on the back from the posterior abdominal wall, and then separating the subcutaneous fat on the back from the surrounding connective tissue after all visceral organs have been removed. Inguinal adipose tissue is the pale-yellow, lobular subcutaneous fat that becomes visible after separating the skin from the muscle layer on the inner thigh of the rat. Perirenal adipose tissue is peeled off from the surface of the kidney after the kidney has been removed. Epididymal adipose tissue is gently separated and removed from the epididymis and vas deferens.

### 2.3. Lipid Analysis of Feces and Liver

Lipid extraction from fecal and liver samples adhered to the methodology described by Tzang et al. [15]. Approximately 0.3 g of homogenized, dried feces or liver tissue was weighed into a glass spiral tube. Seven milliliters of Folch solution (chloroform: methanol = 2:1) were added, followed by thorough homogenization and incubation in an ultrasonic oscillator (DC150H, DELTA^®^, New Taipei City, Taiwan) for 90 min at room temperature. After centrifugation (1250× *g*, 10 °C, 10 min), the supernatant was collected to a fresh glass spiral tube (W0). The solvent was then evaporated using a nitrogen drying system, and the remaining residue was weighed (W1). Total lipid content in both feces and liver tissue was calculated as the difference between W1 and W0. The extracted lipids were subsequently dissolved in isopropanol for analysis of triglycerides (TG) and total cholesterol (TC).

### 2.4. Triglyceride and Total Cholesterol Analysis of Feces and Liver

TG levels were determined using a commercial assay kit from Randox Laboratories Co. Ltd. (Antrim, UK). TC concentrations were assessed with a commercial kit provided by Fortress Diagnostics Co. Ltd. (Antrim, UK).

### 2.5. Perirenal Adipocyte Morphology and Size Evaluation

The morphology and size of perirenal adipocytes were assessed following the method of Galarraga et al. [16]. Perirenal adipose tissue specimens were embedded in paraffin, sectioned, and stained with hematoxylin and eosin (H&E). The stained sections were then viewed under an Olympus BX53 microscope (Tokyo, Japan), and images were captured using an Olympus DP21 two-megapixel digital microscope camera (Tokyo, Japan). The sizes of individual intact adipocytes within the visual field were subsequently quantified using ImageJ (2.42) software.

### 2.6. 16S rRNA Microbial Gene Sequence Analysis

Analysis of the 16S rRNA microbial gene was performed following the protocol detailed by Clarridge [17]. Bacterial DNA was isolated from fecal samples utilizing a commercial DNA extraction kit (Omega Bio-Tek, Norcross, GA, USA). Next-generation sequencing (NGS) was then employed to analyze the highly variable V3-V4 region of the 16S rRNA gene for taxonomic classification. PCR amplification used the forward primer “CAAGCAGAAGACGGCATACGAGAT-[i7]-GTCTCGTGGGCTCGG” and the reverse primer “AATGATACGGCGACCACCGA-GATCTACAC-[i5]-TCGTCGGCAGCGTC”. Adapters were indexed at the ends of the 16S rRNA amplicon, and sequencing was carried out on the Illumina MiSeq platform. Beta-diversity was assessed using unweighted UniFrac distance and principal coordinate analysis (PCoA) to compare the similarity of fecal microbial compositions across different groups. Furthermore, alpha-diversity was analyzed by quantifying microbial composition within each sample, with Simpson and Shannon indexes utilized to estimate microbial diversity using QIIME (version 1) software.

### 2.7. Short-Chain Fatty Acids Analysis of Feces

The measurement of fecal short-chain fatty acids (SCFAs) was conducted following the method described by Kim et al. [18]. In total, 0.02 g of fecal sample was combined with 1000 μL of 2% sulfuric acid, homogenized, and centrifuged at 13,000 rpm for 10 min at 4 °C. The resulting supernatant was filtered through a 0.22 μm syringe filter and transferred into a glass insert for further analysis. The concentrations of acetic acid, propionic acid, and butyric acid were determined using a gas chromatograph Varian CP 3800 equipped with a 30 m × 0.25 mm × 0.25 μm capillary column and a split ratio of 1:50. The assess gas flow rate was set at 1.33 L/min (alone), 31.33 L/min (with nitrogen and hydrogen), and 301 L/min (with air). The column temperature began at 70 °C for 3 min, then increased at a rate of 10 °C/min to 170 °C, where it was held for 22 min. A 1 μL sample was injected at 180 °C, and detection was performed at 200 °C using a flame ionization detector (FID).

### 2.8. Lipid Metabolism Analysis of Liver, WAT, and BAT

For gene expression analysis in various tissues, three rats were selected from each group, chosen based on their final body weights being closest to their respective group averages. Liver, perirenal adipose tissue (white adipose tissue, WAT), and brown adipose tissue (BAT) were collected for this analysis. Total RNA was extracted using a commercial RNA extraction kit (Omega Bio-Tek, Norcross, GA, USA), and complementary DNA (cDNA) was synthesized with a high-capacity cDNA reverse transcription kit (Applied Biosystems, Foster City, CA, USA). For real-time PCR, Power SYBR^®^ Green PCR Master Mix (Applied Biosystems, Foster City, CA, USA) was utilized. The reaction mixture comprised 1 µL of cDNA, 0.6 µL of forward primer (5 µM), 0.6 µL of reverse primer (5 µM), 5 µL of Power SYBR^®^ Green reagent, and 2.8 µL of ddH_2_O. Reactions were carried out on a QuantStudio™ 3 Real-Time PCR System (Applied Biosystems, Foster City, CA, USA). Relative gene expression levels were normalized using β-actin as the housekeeping gene. The primer sequences employed for real-time PCR were as follows: *β-actin*, 5′-TACAATGAGCTGCGTGTGG-3′ (forward) and 5′-TGGTGGTGAAGCTGTAGCC-3′ (reverse); *PPAR-α*, 5′-CATCGAGTGTCGAATATGTGG-3′ (forward) and 5′-GCAGTACTGGCATTTGTTCC-3′ (reverse); *CPT-1*, 5′-GCTCGCACATTACAAGGACAT-3′ (forward) and 5′-TGGACACCACATAGAGGCAG-3′ (reverse); *PPAR-γ*, 5′-CATGACCAGGGAGTTCCTCAA-3′ (forward) and 5′-AGCAAACTCAAACTTAGGCTCCAT-3′ (reverse); *FATP-1*, 5′-GTGCGACAGATTGGCGAGTT-3′ (forward) and 5′-GCGTGAGGATACGGCTGTTG-3′ (reverse); *SIRT-1*, 5′-GATCTCCCAGATCCTCAAGCC-3′ (forward) and 5′-CACCGAGGAACTACCTGAT-3′ (reverse); *FAS*, 5′-CTTGGGTGCCGATTACAACC-3′ (forward) and 5′-GCCCTCCCGTACACTCACTC-3′ (reverse); and *PGC-1α*, 5′-CAATGAGCCCGCGAACATAT-3′ (forward) and 5′-CAATCCGTCTTCATCCACCG-3′ (reverse).

### 2.9. Statistical Analysis

All experimental data underwent analysis using Sigma Plot software (version 10.0). Group comparisons for differences were performed via an unpaired *t*-test, and results are presented as the mean ± standard error (SEM). For assessing alpha-diversity within fecal microbiota, data were processed using QIIME (version 1) software. Unpaired *t*-tests were used for two-group comparisons, while Kruskal–Wallis tests assessed multi-group alpha-diversity; one-way ANOVA will be considered for future multi-group analyses to enhance statistical robustness. A *p*-value below 0.05 was consistently considered to indicate statistical significance for all analyses.

## 3. Results

### 3.1. EQR’s Impact on Growth Metrics in Obese HFD-Fed Rats

**Figure 1:**
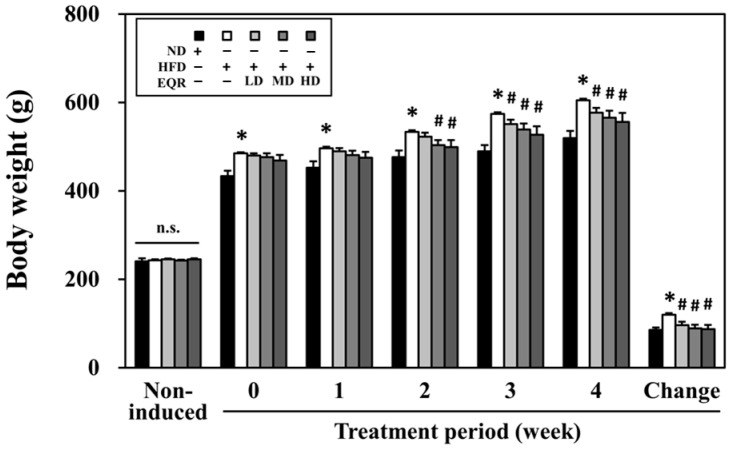
Effect of EQR on the body weight in obese HFD-fed rats. The reported values were the mean ± SEM (*n* = 10). * *p* < 0.05 compared to the ND group. # *p* < 0.05 compared to the HFD group. N.S., no significant differences; ND, normal diet; HFD, high-fat diet; EQR, epigallocatechin-3-gallate-quercetin-rutin compounds; LD, low-dose; MD, medium-dose; HD, high-dose. Body weight change (g) = weight of week 4 − weight of week 0.

The progression of rat body weights throughout the experiment is illustrated in Figure 1. Following four weeks of high-fat diet exposure, the HFD group’s body weight was 10% greater than that of the ND group. In addition, the results showed that medium-dose (MD) and high-dose (HD) EQR significantly reduced the body weights in HFD groups during the second week of treatment. In the third week of treatment, rats in all EQR-treated groups displayed significantly lower body weights compared to the HFD control group. A high-fat diet exhibited significantly lower feed intake but higher energy intake and feed efficiency compared to those rats on a normal diet. Particularly, EQR treatment at all three doses significantly reduced feed efficiency, while water intake remained comparable across all groups (Table 1).

### 3.2. Effects of EQR on Adipose Tissues in Obese HFD-Fed Rats

**Figure 2:**
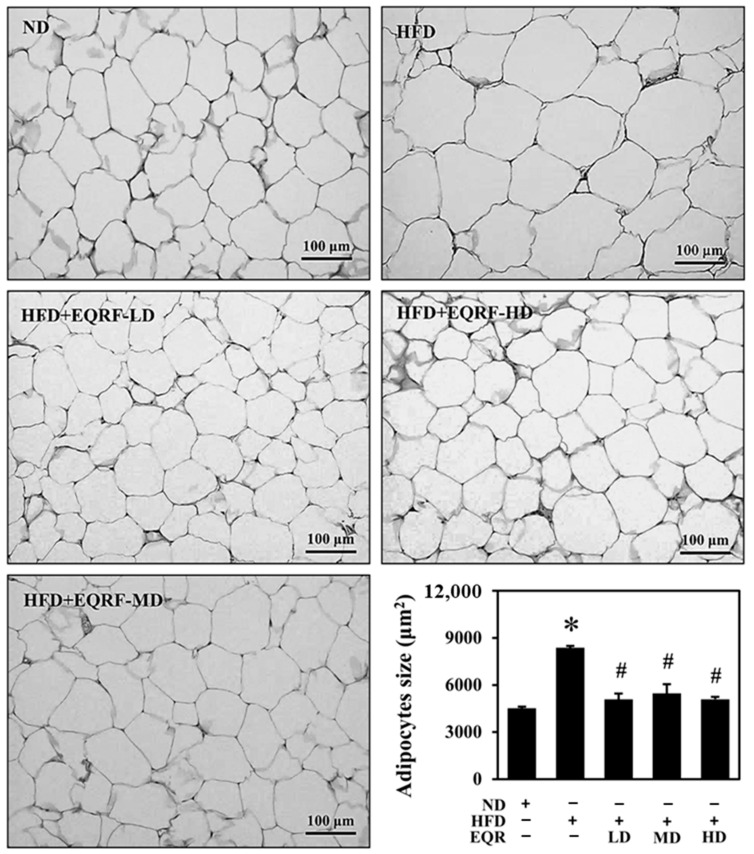
Effect of EQR on the morphology and size of perirenal adipocytes in obese HFD-fed rats. The perirenal adipose tissues were stained with hematoxylin and eosin (H&E). Original magnification: 200×. The reported values were the mean ± SEM (*n* = 6). * *p* < 0.05 compared to the ND group. # *p* < 0.05 compared to the HFD group. ND, normal diet; HFD, high-fat diet; EQR, epigallocatechin-3-gallate-quercetin-rutin compounds; LD, low-dose; MD, medium-dose; HD, high-dose.

All measured adipose tissue depots were considerably heavier in the HFD control group than in the ND group, a condition mitigated by EQR treatment. All EQR-treated groups exhibited a marked decrease in visceral adipose tissue weight. Specifically, the EQR-LD group exhibited a significant decrease in perirenal adipose tissue, the EQR-MD group showed reductions in both perirenal and mesenteric adipose tissues, and the EQR-HD group demonstrated significant decreases across all three visceral fat depots. For subcutaneous adipose tissues, the EQR-LD group did not display any significant differences in comparison with the HFD control group, while both the EQR-MD and EQR-HD groups displayed significantly lower weights of subcutaneous and inguinal adipose tissues. Additionally, all three EQR doses significantly reduced total body fat mass (Table 1). Based on the significance of the above results, we proceeded with H&E staining of the perirenal adipose tissue to assess the impact on morphology and size (Figure 2). The results demonstrated that the adipocytes exhibited significantly enlarged adipocytes in the HFD control group compared with that in the ND group. Conversely, adipocytes in all three EQR treatment groups appeared smaller than those in the HFD control group.

### 3.3. Effects of EQR on the Hepatic and Fecal Lipid Levels in Obese HFD-Fed Rats

Table 2 shows the lipid levels of the feces and liver after EQR treatment for 4 weeks. The HFD control group exhibited significantly higher hepatic total lipid, hepatic TG, fecal total lipid, and fecal TC levels compared to the ND group. EQR treatments significantly reduced hepatic total lipid levels in both the EQR-MD and EQR-HD groups. However, no significant differences were observed among groups in hepatic TC and fecal TG levels.

### 3.4. Effects of EQR on the Short-Chain Fatty Acids Contents in HFD-Induced Obese Rats

**Figure 3:**
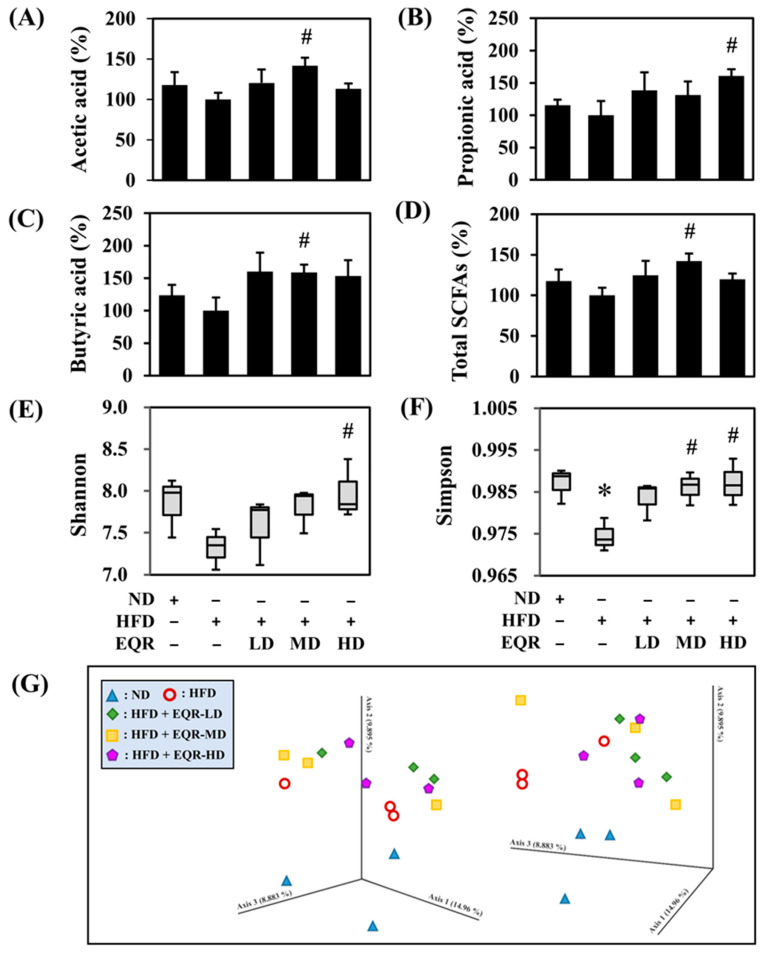
Effect of EQR on the content of SCFAs; the diversity and compositions of gut microbiota in obese HFD-fed rats. SCFA contents, (**A**) Acetic acid, (**B**) Propionic acid, (**C**) Butyric acid, and (**D**) Total SCFAs. The reported values of SCFA contents were the mean ± SEM of the relative percent of HFD (*n* = 6). Alpha diversity, (**E**) Shanon and (**F**) Simpson. Beta diversity, (**G**) Principal coordinate analysis (PCoA). The boxplot demonstrated a distribution summary of diversity indices estimated at the OUT level (*n* = 3). PCoA of fecal microbial compositions was shown, which was based on the unweighted UniFrac distance measure of all samples based on relative abundance profiles of the OUT level (*n* = 3). * *p* < 0.05 compared to the ND group. # *p* < 0.05 compared to the HFD group. ND, normal diet; HFD, high-fat diet; EQR, epigallocatechin-3-gallate-quercetin-rutin compounds; LD, low-dose; MD, medium-dose; HD, high-dose.

Acetic acid (Figure 3A), propionic acid (Figure 3B), butyric acid (Figure 3C), and total SCFAs (Figure 3D) were quantified in fecal SCFAs. The SCFA profile showed acetic acid (~85%), propionic acid (~7%), and butyric acid (~8%) in the ND group. Although the HFD control group presented a marginal decline in all SCFA levels in comparison to the ND group. EQR supplementation significantly increased fecal acetic acid, butyric acid, and total SCFA levels in the EQR-MD group as well as the EQR-HD group and also caused a considerable rise in propionic acid levels.

### 3.5. Effects of EQR on Gut Microbial Composition in Obese HFD-Fed Rats

**Figure 4:**
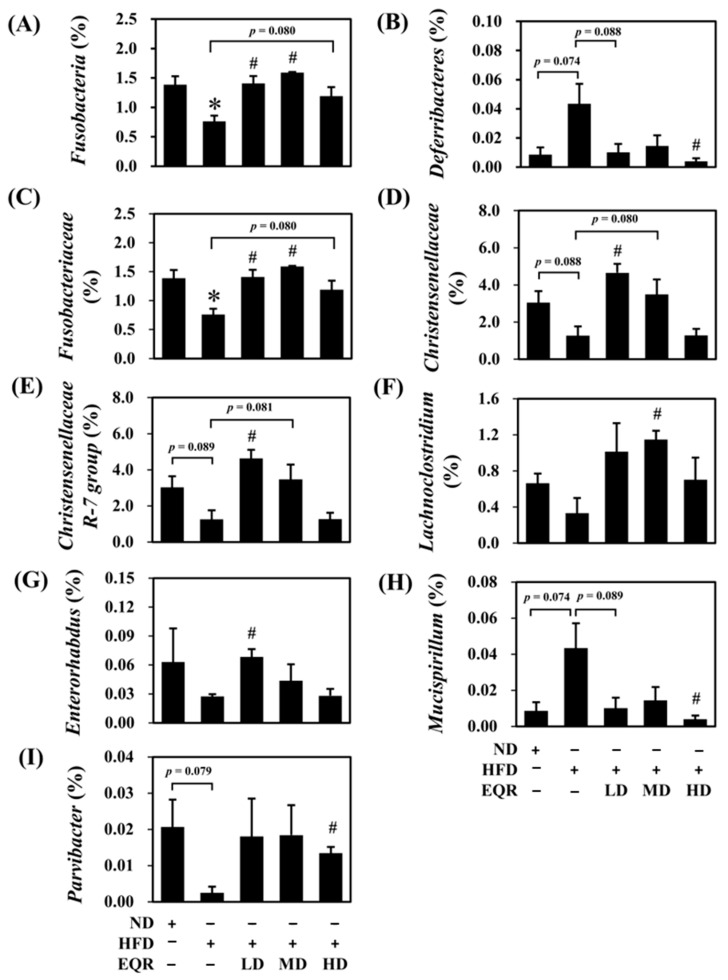
Effect of EQR treatment on the abundance of gut microbial compositions in obese HFD-fed rats. The phylum level, (**A**) *Fusobacteria* and (**B**) *Deferribacteres*. The family level, (**C**) *Fusobacteriaceae* and (**D**) *Christensenellaceae*. The genus level, (**E**) *Christensenellaceae R-7 group*, (**F**) *Lachnoclostridium*, (**G**) *Enterorhabdus*, (**H**) *Mucispirillum*, and (**I**) *Parvibacter*. The reported values are the mean ± SEM (*n* = 3). * *p* < 0.05 compared with the ND group. # *p* < 0.05 compared with the HFD group. ND, normal diet; HFD, high-fat diet; EQR, epigallocatechin-3-gallate-quercetin-rutin compounds; LD, low-dose; MD, medium-dose; HD, high-dose.

The diversity in gut microbiota was considered using the α-diversity analysis (Shanon and Simpson indexes), and the reported data are presented in a quartile boxplot (Figure 3E,F). Simpson indexes indicated that the diversity in the gut microbiota decreased after HFD induction. Simpson indices were markedly increased in both the EQR-MD and EQR-HD groups, restoring microbial diversity. Additionally, the HD group displayed a notable elevation in the Shannon index, reflecting enhanced and richness evenness of the gut microbial community. β-diversity analysis was performed using principal coordinates analysis (PCoA) to assess changes in gut microbiota composition across groups (Figure 3G). The PCoA results indicated distinct clustering among the ND, HFD, and EQR-treated groups, which demonstrated that EQR treatment regulated the gut microbiota composition. Specifically, the gut microbiota of all EQR-treated groups (LD, MD, and HD) demonstrated a clear separation from the HFD group and a shift toward the ND group that suggested partial restoration of a healthier microbial community. The gut microbiota is predominantly composed of the bacterial phyla *Firmicutes*, *Bacteroidetes*, *Fusobacteria*, *Actinobacteria*, and *Proteobacteria*, with *Firmicutes* and *Bacteroidetes* constituting over 90% of the total bacterial groups. The HFD group showed slightly increased abundances of *Firmicutes* and slightly decreased abundances of *Bacteroidetes* compared to the ND group (*p* > 0.05). EQR treatment did not significantly reverse this tendency. Furthermore, no statistically significant differences were detected in the abundance of *Actinobacteria* and *Proteobacteria* among the groups (*p* > 0.05). The relative abundance of *Fusobacteria* markedly diminished in the HFD group (*p* < 0.05). Conversely, EQR-MD and EQR-HD treatments improved its abundance to a level comparable with the ND group at the phylum level (Figure 4A). *Deferribacteres* showed a marginal increase in the HFD control group (*p* = 0.074), and a significant decrease in the EQR-HD group (*p* < 0.05) (Figure 4B). At the family level, the abundance of *Fusobacteriaceae* significantly reduced with HFD and increased with low-dose and medium-dose EQR intervention (Figure 4C). In contrast, *Christensenellaceae* levels slightly decreased in the HFD control group, with a significant increase observed after EQR-LD treatment (Figure 4D). At the genus level, *Christensenellaceae R-7 group* abundance decreased marginally in the HFD group (*p* = 0.089), and increased significantly after low-dose EQR treatment (Figure 4E). Similarly, a high-fat diet slightly reduced *Lachnoclostridium* levels, which significantly increased following medium-dose EQR intervention (*p* < 0.05) (Figure 4F), while *Enterorhabdus* levels were also markedly elevated in the EQR-MD group (*p* < 0.05) (Figure 4G). Moreover, *Mucispirillum* levels marginally increased in the HFD group (*p* = 0.074), reducing significantly with EQR-HD supplementation (Figure 4H). Finally, *Parvibacter* exhibited a slight reduction in the HFD group (*p* = 0.079), while a significant induction in the EQR-HD group (Figure 4I).

### 3.6. Effects of EQR on Lipid Metabolism in Obese HFD-Fed Rats

**Figure 5:**
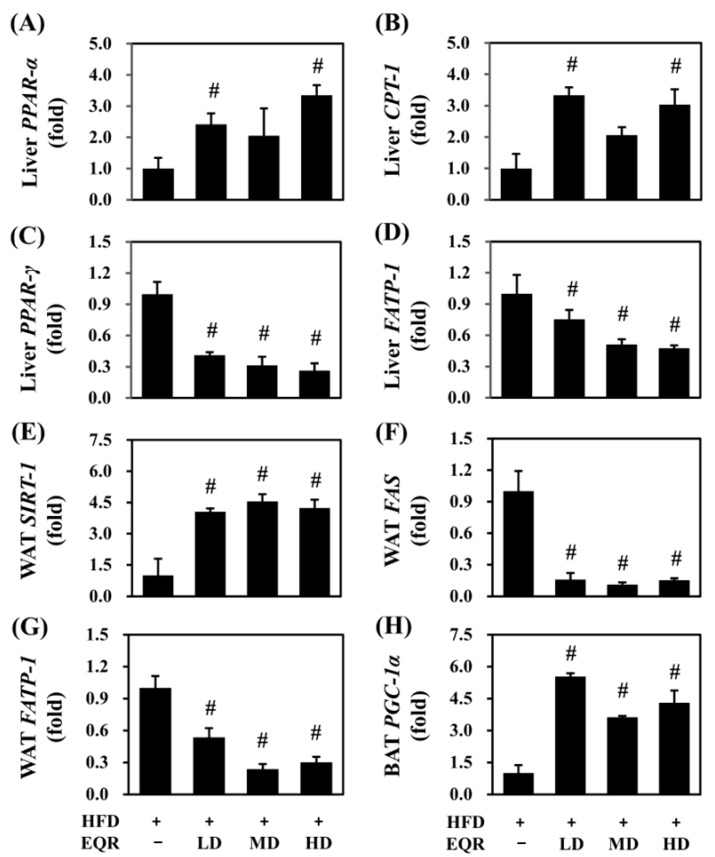
Effect of EQR on the lipid metabolism in obese HFD-fed rats. (**A**) Liver *PPAR-α*, (**B**) Liver *CPT-1*, (**C**) Liver *PPAR-γ*, (**D**) Liver *FATP-1*, (**E**) WAT *SIRT-1*, (**F**) WAT *FAS*, (**G**) WAT *FATP-1*, and (**H**) BAT *PGC-1α*. The reported values were the mean ± SEM of the relative mRNA levels (*n* = 3). # *p* < 0.05 compared to the HFD group. ND, normal diet; HFD, high-fat diet; EQR, epigallocatechin-3-gallate-quercetin-rutin compounds; LD, low-dose; MD, medium-dose; HD, high-dose.

Based on the observed anti-obesity effects of EQR, we further investigated the underlying mechanisms in HFD-induced obese rats by analyzing the relative mRNA expression levels in the liver tissue, brown adipose tissue (BAT), and white adipose tissue (WAT). Low-dose and high-dose EQR treatment markedly elevated hepatic *PPAR-α* and *CPT-1* levels (*p* < 0.05) (Figure 5A,B), indicating enhanced fatty acid oxidation. Meanwhile, *PPAR-γ* level was significantly reduced in all EQR groups (*p* < 0.05) (Figure 5C), and *FATP-1* was markedly downregulated in all EQR-treated groups (*p* < 0.05) (Figure 5D), suggesting suppressed lipid synthesis and uptake. In WAT, *SIRT-1* expression levels were notably elevated (*p* < 0.05) (Figure 5E), while both *FAS* and *FATP-1* were notably decreased after EQR treatment (*p* < 0.05) (Figure 5F,G), indicating inhibition of lipogenesis. In BAT, EQR supplementation at all three doses significantly upregulated *PGC-1α* (*p* < 0.05) (Figure 5H), suggesting promoted energy expenditure.

## 4. Discussion

Diet-induced obesity models, particularly relevant to human obesity development, are commonly used in metabolic studies. Despite the absence of precise criteria for the diagnosis of animal obesity, researchers typically assess it using markers, including body weight, adipose tissue weight, and adipocyte size, comparing these to control animals, [19]. In our study, rats were fed a high-fat diet to induce obesity, followed by three doses of EQR treatment. The results showed that EQR treatment significantly reduced body weight and feed efficiency without altering energy intake. It also decreased adipose tissue weight, adipocyte size, and liver lipid content caused by the HFD. Previous studies indicated that EGCG (25 and 50 mg/kg for 8 weeks) reduced final body weight, liver weight, and hepatic lipid accumulation in HFD-fed mice [20]. Similarly, an 8-week treatment with a quercetin-rich supplement (1, 2, and 5 servings; 1 serving = 185 mg/kg) decreased final body weight, body fat, liver weight, and adipocyte size, while increasing urinary lipid content in HFD-fed rats [13]. Rutin (50 and 100 mg/kg) also showed anti-obesity effects, reducing body weight, adipose tissue weight, and liver lipid content in HFD-fed rats over 8 weeks [21]. Furthermore, a synergistic anti-obesity effect on inflammation and gut flora was observed when quercetin was combined with resveratrol (45, 90, and 108 mg/kg at a 2:1 ratio) in obese rats over 8 weeks [22]. Our results showed that the administration of a low dose of EQR could decrease the final body weight, visceral adipose tissue weight, and adipocyte size in obese rats, revealing a lower usage amount and treatment period compared with these other studies. This suggests a potentially higher anti-obesity efficiency for these combined compounds, likely due to a synergistic effect.

A High-Fat Diet resulted in the accumulation of excessive fat in the body that alters gut microbiota composition. Diets rich in fat and protein or low in fiber can trigger systemic inflammation, gut barrier dysfunction (“leaky mucosa”), and decreased production of SCFAs and enteroendocrine hormones, which can cooperatively reduce energy expenditure [23]. Our finding revealed that EQR treatment effectively increased the SCFA contents in the gut. Acetic acid is the most abundant (approximately 57%) and plays a role in cholesterol synthesis. Propionic acid is a valuable metabolic precursor for the synthesis of glucose, lipids, and proteins, and studies show it can reduce fat accumulation in the liver and visceral areas. Butyric acid is crucial as the primary energy source for the colonic epithelium, supporting colon health and mucosal proliferation to defend against harmful substances [24,25]. Mansoorian et al. (2019) show that oral polyphenols are generally poorly absorbed, meaning a substantial amount reaches the colon where they are metabolized by the resident microbiota [26]. The antioxidant, prebiotic, and antimicrobial properties of EQR’s polyphenols are likely responsible for its modulation of the gut microbiota [27,28]. The increase in beneficial taxa such as *Fusobacteria*, *Christensenellaceae*, *Lachnoclostridium*, *Enterorhabdus*, and *Parvibacter* may be attributed to the prebiotic-like effects of EGCG, quercetin, and rutin, which serve as substrates for microbial fermentation, promoting the growth of SCFA-producing bacteria. A previous study showed that a 12-week administration of an HFD with 300 mg/kg of pomegranate peel polyphenols not only reduced body weight and liver lipid accumulation in rats but also increased SCFA content by promoting SCFA-producing bacteria such as *Lactobacillus*, *Roseburia*, and the *Christensenellaceae R-7 group* [29]. Another study showed that a 12-week treatment of HFD-fed mice with 100 and 200 mg/kg of phenolic-rich Rhodomyrtus tomentosa fruit extract decreased body weight and increased urinary SCFA content. This was achieved by promoting SCFA producers and degrading intestinal mucosal mucin, thus preserving the intestinal barrier and preventing HFD-induced metabolic disorders [30]. Our study suggests that EQR treatment enhances SCFA production through a significant increase in SCFA-producing bacteria, thereby protecting the intestinal barrier and mitigating the detrimental effects of HFD.

According to the present results, EQR exhibited the potential to improve the gut microbiota composition. Specifically, the phylum *Deferribacteres* increased in rats fed an HFD. Previous reports demonstrated that *Deferribacteres* associated with inflammatory factors such as IL-6 and TNF-α. Critically, with reduced abundance of *Deferribacteres* shown to alleviate HFD-induced chronic inflammation [31,32], *Fusobacteria* was one of the butyrate producers [33,34]. Our research observed HFD-induced decreases in both *Fusobacteria* and butyric acid, which EQR treatment reversed. At the family level, *Christensenellaceae* and *Fusobacteriaceae* were depleted in HFD groups but restored by EQR. *Christensenellaceae* are associated with lean individuals and reduced visceral fat [35,36]. *Fusobacteriaceae* also played a vital role in normal-weight subjects [37]. Tamura et al. (2017) showed that the abundance of *Fusobacteriaceae* was positively correlated with the fecal quercetin level, with the results suggesting that *Fusobacteriaceae* might inhibit other bacteria from breaking down quercetin [38]. At the level of genus, *Mucispirillum* increased in the rats fed with an HFD in our report. The bacteria were negatively correlated with body weight, total cholesterol, and blood glucose [39,40]. Conversely, beneficial genera such as the *Christensenellaceae R-7 group*, *Lachnoclostridium*, *Enterorhabdus*, and *Parvibacter* decreased in HFD-fed rats. These bacteria, abundant in normal-weight hosts, function as SCFA producers and inflammation suppressors. They further contribute to health by promoting lipolysis and fat oxidation, improving insulin sensitivity and counteracting cholesterol synthesis [40,41,42,43]. Thus, EQR’s ability to restore gut microbial dysbiosis mitigated the negative effects of a high-fat diet.

Connecting our observations on obesity and gut microbiota, we elucidated the mechanism of EQR in HFD-induced rats. Previously, we identified that an HFD markedly downregulated fatty acid synthesis and upregulated β-oxidation in both liver and adipose tissues [13]. Our current investigation revealed that EQR treatment enhanced fatty acid oxidation by acting downstream of the AMPK pathway. *CPT-1* was a vital modulator of β-oxidation in hepatic mitochondrial, transformed fatty acyl-CoA into fatty acylcarnitine, and promoted fatty acid metabolism. Similarly, the nuclear transcription factors *PPARα* and *PPARγ* are critical for lipid metabolism. In the present findings, HFD induction decreased *PPARα* and increased *PPARγ* levels; EQR effectively reversed these imbalances [44]. The upregulation of *PPAR-α* and *CPT-1* in the liver following EQR treatment signifies enhanced fatty acid oxidation, a critical mechanism for reducing hepatic lipid accumulation in obesity. Moreover, we found that EQR modulated *FATP1*, which is responsible for absorbing and accumulating free fatty acids in tissues such as the liver, muscles, and adipose tissue [45]. Our study found that EQR significantly reduced *FATP1* mRNA expression in the liver and white adipose tissue, which correlated with decreased liver lipid levels and adipocyte size. Furthermore, In obese rats, *SIRT-1*, a critical regulator of cellular energy balance, is often inhibited, while *FAS* is stimulated, leading to excess lipid accumulation and liver steatosis [46]; EQR treatment effectively downregulated *PPAR-γ* and *FATP-1*, indicating suppressed lipid synthesis and uptake. This finding corroborates the observed decrease in liver lipid content. In white adipose tissue, increased *SIRT-1* and decreased *FAS*/*FATP-1* expression suggest the inhibition of lipogenesis, which aligns with smaller adipocytes. Additionally, our results indicated that EQR treatment improved the expression of *PGC-1α* in BAT. BAT exhibited thermogenesis through the uncoupling of mitochondrial oxidation, which produced ATP and energy [47]. *PGC-1α* is a critical thermogenic gene that regulates mitochondrial metabolism. The increased *PGC-1α* in brown adipose tissue highlights enhanced thermogenesis, contributing to the overall anti-obesity effect. These results suggested induced fatty acid synthesis and reduced β-oxidation in the liver and adipocyte tissues after EQR treatment compared with those in the HFD groups.

This study did not explicitly discuss its limitations. Potential biases include the lack of detailed randomization and blinding methods. Animal model limitations arise from using only male Wistar rats, which may limit generalizability. Imprecision could stem from the unstated sample size calculation, potentially affecting the detection of subtle effects within the four-week intervention period. In this study, we evaluated the anti-obesity effect of the EQR supplement using three distinct doses. Low doses of the EQR intervention significantly decreased the body weight, visceral adipose tissue weight, and total body fat, while medium and high doses reduced the subcutaneous adipose tissue weight and liver lipid content. Interestingly, despite these specific effects, there were no significant overall differences in anti-obesity outcomes found among the intervention groups. This suggests a plateau effect, likely due to the potent synergistic action of the three polyphenol compounds in EQR, achieving maximum anti-obesity benefits even at lower concentrations.

## 5. Conclusions

In conclusion, EQR treatment effectively reduced body weight gain, feed efficiency, adipose tissue weight, and liver lipid content in HFD-induced obese rats. It also enhanced SCFA levels and positively modulated gut microbiota diversity and composition. The low dose (103 mg/kg) was sufficient to elicit significant anti-obesity effects, including reduced visceral adipose tissue and body fat, while medium (206 mg/kg) and high (514 mg/kg) doses further decreased subcutaneous adipose tissue and liver lipid content. This study reveals that EQR’s synergistic low-dose formulation outperforms individual high-dose treatments by rapidly modulating gut microbiota and lipid metabolism, presenting a novel strategy for obesity management.

## Figures and Tables

**Table 1:** Effects of EQR on growth parameters and adipose tissues weight in obese HFD-fed rats.

Items	ND	HFD			
		Control	EQR-LD	EQR-MD	EQR-HD
Growth parameters					
Feed intake (g/rat/day)	33.31 ± 0.94	23.22 ± 0.37 *	22.43 ± 0.49	22.40 ± 0.68	21.83 ± 0.74
Energy intake (kcal/rat/day)	100.07 ± 2.80	117.26 ± 1.84 *	113.27 ± 2.49	112.05 ± 3.42	110.13 ± 3.80
Feed efficiency (%)	13.47 ± 0.57	27.19 ± 0.55 *	22.51 ± 1.49 ^#^	20.76 ± 1.45 ^#^	20.65 ± 1.78 ^#^
Water intake (mL/rat/day)	36.79 ± 2.39	39.48 ± 1.99	47.61 ± 4.22	44.66 ± 3.39	42.02 ± 3.07
Adipose tissue weight (mg/g rat)					
Total body fat	98.72 ± 6.19	138.99 ± 4.98 *	114.36 ± 7.59 ^#^	104.53 ± 6.79 ^#^	106.04 ± 8.89 ^#^
Subcutaneous	27.39 ± 1.94	42.24 ± 2.48 *	34.15 ± 3.40	30.00 ± 2.46 ^#^	31.27 ± 3.26 ^#^
Visceral	71.16 ± 4.93	96.99 ± 3.83 *	79.94 ± 4.58 ^#^	74.54 ± 5.52 ^#^	74.45 ± 6.08 ^#^
Mesenteric	17.39 ± 1.55	22.85 ± 1.56 *	19.63 ± 1.52	16.18 ± 1.56 ^#^	16.54 ± 1.32 ^#^
Retroperitoneal	15.99 ± 1.48	22.99 ± 1.17 *	20.81 ± 2.74	18.50 ± 2.48	19.06 ± 2.58
Inguinal	11.45 ± 1.19	19.12 ± 2.38 *	13.53 ± 1.46	11.52 ± 1.32 ^#^	12.41 ± 1.91 ^#^
Perirenal	30.03 ± 2.72	42.51 ± 1.99 *	32.61 ± 2.59 ^#^	32.16 ± 2.95 ^#^	31.54 ± 3.26 ^#^
Epididymal	23.53 ± 1.64	31.78 ± 1.70 *	27.52 ± 1.41	26.27 ± 2.31	26.12 ± 1.86 ^#^

The reported values were the mean ± SEM (*n* = 10). * *p* < 0.05 compares to the ND group. ^#^ *p* < 0.05 compared to the HFD group. ND, normal diet. HFD, high-fat diet. EQR, epigallocatechin-3-gallate-quercetin-rutin. LD, low-dose. MD, medium-dose. HD, high-dose. Feed efficiency (%) = [Body weight change (g)/total feed intake (g)] × 100%. Total body fat (mg/g rat) = [visceral adipose tissue (mg) + subcutaneous adipose tissue (mg)]/final body weight (g). Subcutaneous adipose tissue (mg/g rat) = [retroperitoneal adipose tissue (mg) + inguinal adipose tissue (mg)]/final body weight (g). Visceral adipose tissue (mg/g rat) = [perirenal adipose tissue (mg) + epididymal adipose tissue (mg) + mesenteric adipose tissue (mg)]/final body weight (g).

**Table 2:** Effects of EQR on liver and feces lipid contents in obese HFD-fed rats.

Items	ND	HFD			
		Control	EQR-LD	EQR-MD	EQR-HD
Liver (mg/g tissue)					
Total lipid	72.50 ± 1.94	119.92 ± 10.01 *	124.12 ± 10.57	92.85 ± 5.35 ^#^	92.33 ± 5.68 ^#^
Triglyceride	14.29 ± 1.39	33.15 ± 3.13 *	39.92 ± 4.48	28.65 ± 2.94	27.36 ± 3.09
Total cholesterol	6.45 ± 0.05	7.53 ± 0.85	6.48 ± 0.06	6.44 ± 0.03	6.49 ± 0.04
Feces (mg/g dried feces)					
Total lipid	28.93 ± 2.28	40.44 ± 2.63 *	37.39 ± 1.77	44.01 ± 2.59	44.43 ± 2.02
Triglyceride	3.75 ± 0.21	5.00 ± 1.23	5.23 ± 1.21	6.69 ± 1.68	5.67 ± 1.30
Total cholesterol	3.05 ± 0.10	3.95 ± 0.23 *	4.08 ± 0.38	4.27 ± 0.34	3.60 ± 0.25

The reported values were the mean ± SEM (*n* = 10). * *p* < 0.05 compares to the ND group. ^#^ *p* < 0.05 compared to the HFD group. ND, normal diet; HFD, high-fat diet; EQR, epigallocatechin-3-gallate-quercetin-rutin compounds; LD, low-dose; MD, medium-dose; HD, high-dose.

## Data Availability

The data presented in this study are available on request from the corresponding author. The data is publicly available.
